# Lifestyle behaviour in adolescence and musculoskeletal pain 11 years later: The Trøndelag Health Study

**DOI:** 10.1002/ejp.2012

**Published:** 2022-08-26

**Authors:** Kaja Smedbråten, Margreth Grotle, Henriette Jahre, Kåre Rønn Richardsen, Milada Cvancarova Småstuen, Eva Skillgate, Britt Elin Øiestad

**Affiliations:** ^1^ Department of Physiotherapy Oslo Metropolitan University Oslo Norway; ^2^ Research and Communication Unit for Musculoskeletal Health (FORMI), Division of Clinical Neuroscience Oslo University Hospital Oslo Norway; ^3^ Musculoskeletal & Sports Injury Epidemiology Center Sophiahemmet University Stockholm Sweden; ^4^ Institute of Environmental Medicine, Karolinska Institutet Stockholm Sweden

## Abstract

**Background:**

There is limited knowledge on the association between lifestyle behaviour in adolescence and musculoskeletal pain in young adulthood. This study aimed to investigate whether an accumulation of adverse lifestyle behaviours in adolescents with and without musculoskeletal pain at baseline, was associated with persistent musculoskeletal pain (pain duration ≥3 consecutive months the last year) 11 years later.

**Methods:**

Longitudinal data from the Trøndelag Health Study in Norway including 1824 adolescents (13–19 years old) was analysed. The outcome was persistent musculoskeletal pain (≥3 months). The number of adverse lifestyle behaviours (low physical activity level, sleep problems, insufficient fruit/vegetables consumption, smoking, frequent alcohol intoxication [drunkenness] and/or illicit drug use) were summed up to comprise an ordinal variable and analysed with 0 or 1 adverse behaviours as the reference. Multiple logistic regression analyses, stratified by individuals with and without baseline musculoskeletal pain, were conducted. The results were expressed as odds ratios (ORs) with 95% confidence intervals (CIs).

**Results:**

In adolescents with musculoskeletal pain at baseline, reporting ≥ four adverse lifestyle behaviours increased the odds of persistent musculoskeletal pain (OR 2.23, 95% CI 1.36, 3.66) 11 years later. Two and three adverse behaviours were not associated with future persistent musculoskeletal pain. In adolescents without musculoskeletal pain at baseline, an accumulation of adverse lifestyle behaviours was not associated with future persistent musculoskeletal pain.

**Conclusion:**

An accumulation of adverse lifestyle behaviours in adolescents with musculoskeletal pain at baseline was associated with persistent musculoskeletal pain 11 years later, but not in adolescents without musculoskeletal pain at baseline.

**Significance:**

An accumulation of four or more adverse lifestyle behaviours in adolescents with musculoskeletal pain was associated with persistent musculoskeletal pain in young adulthood. In future health care of adolescents with musculoskeletal pain, lifestyle behaviours should be assessed, with emphasis on accumulation of multiple adverse lifestyle behaviours. Focusing on an accumulation of multiple adverse lifestyle behaviours, rather than each individual behaviour, might provide a potential area for future research and interventions targeting musculoskeletal pain in youth.

## 1 INTRODUCTION

Persistent musculoskeletal pain (≥3 months duration) is prevalent already in adolescence and young adulthood (Hagen et al., 2011; Malmborg et al., 2019). In 15–19‐year‐olds and 20‐24‐year‐olds, musculoskeletal pain is the fourth and second leading cause of years lived with disability, respectively, and from 25 years of age, it is the leading cause (Vos et al., 2016). Furthermore, experiencing musculoskeletal pain in young ages increases the risk of later episodes (Jeffries et al., 2007; Øiestad et al., 2020). Consequently, the knowledge of early modifiable risk and prognostic factors is important to design intervention studies and potentially prevent future persistent musculoskeletal pain.

Systematic reviews of longitudinal studies on children and adolescents have reported that smoking might increase the risk of musculoskeletal pain, while body mass index (BMI) probably does not (Huguet et al., 2016). Sleep problems might increase the risk of pain in some body regions and subgroups, but not for musculoskeletal pain in general (Andreucci et al., 2017). For physical activity level, results from a systematic review of longitudinal and cross‐sectional studies indicated that both very high and very low activity levels were associated with low back pain in children and adolescents (Kędra et al., 2021). For alcohol consumption and future low back pain, the results from longitudinal studies are inconsistent (Hestbaek et al., 2006a; Smith et al., 2017), while there are few studies examining the association between other lifestyle behaviours in adolescence, such as diet and illicit drug use, and future musculoskeletal pain. For young adults (18‐29 years of age), there is conflicting evidence on associations between physical activity, smoking, BMI and future back pain (Øiestad et al., 2020), and no associations have been reported for either physical activity or BMI with future neck pain (Jahre et al., 2020). In adolescents with musculoskeletal pain, a systematic review reported that smoking and sleep‐related problems have been found as potential prognostic factors across two and five cohort studies, respectively (Pourbordbari et al., 2019).

Lifestyle behaviours often cluster (Uddin et al., 2020). As the existing literature on individual lifestyle behaviours and musculoskeletal pain is inconsistent, investigating the cluster effect, i.e. of multiple adverse lifestyle behaviours, might be beneficial. Longitudinal studies on adults have indicated that healthy levels of physical activity and fruit/vegetables, low alcohol intake and no smoking combined, protected against incident back pain (Pronk et al., 2010), long‐duration troublesome low back pain in men, and long‐duration troublesome neck pain in women (Skillgate et al., 2017). Furthermore, a combination of healthy behaviours influenced the prognosis of neck pain (Bohman et al., 2019) and low back pain in women (Bohman et al., 2014). In adolescents, cross‐sectional analyses of the present study's baseline data showed an association between an accumulation of adverse lifestyle behaviours and persistent pain (≥3 months) (Hoftun et al., 2012). Evidence of whether multiple adverse lifestyle behaviours in adolescence increases the long‐term risk of musculoskeletal pain is, however, lacking.

The aim of this study was to investigate whether an accumulation of adverse lifestyle behaviours, including low physical activity level, sleep problems, insufficient fruit/vegetables consumption, smoking, frequent alcohol intoxication (drunkenness) and/or illicit drug use, in adolescents with and without musculoskeletal pain at baseline, was associated with persistent musculoskeletal pain (pain duration ≥3 consecutive months the last year) 11 years later.

## 2 METHODS

### 2.1 Study design and sample

Data for this cohort study was obtained from a large population‐based study in Norway; the Trøndelag Health Study (HUNT) (Åsvold et al., 2022; Holmen et al., 2013). Data from the third adolescent survey (Young‐HUNT3) collected from 2006 to 2008, and the fourth adult survey (HUNT4) collected 11 years later (2017–2019) were used. All inhabitants aged 13–19 years living in Nord‐Trøndelag were invited to participate in Young‐HUNT3. The participants were recruited through the local schools, and those who consented to participate completed a comprehensive questionnaire during school hours. Participants were invited to a subsequent clinical examination, including measurements of weight and height. Adolescents not attending school were contacted by mail and invited to attend the examination at the nearest field station. An invitation letter to participate in the next survey (HUNT4) was sent by mail, and the participants were asked to complete an electronic questionnaire and to undertake physical tests at a local field station.

**FIGURE 1:**
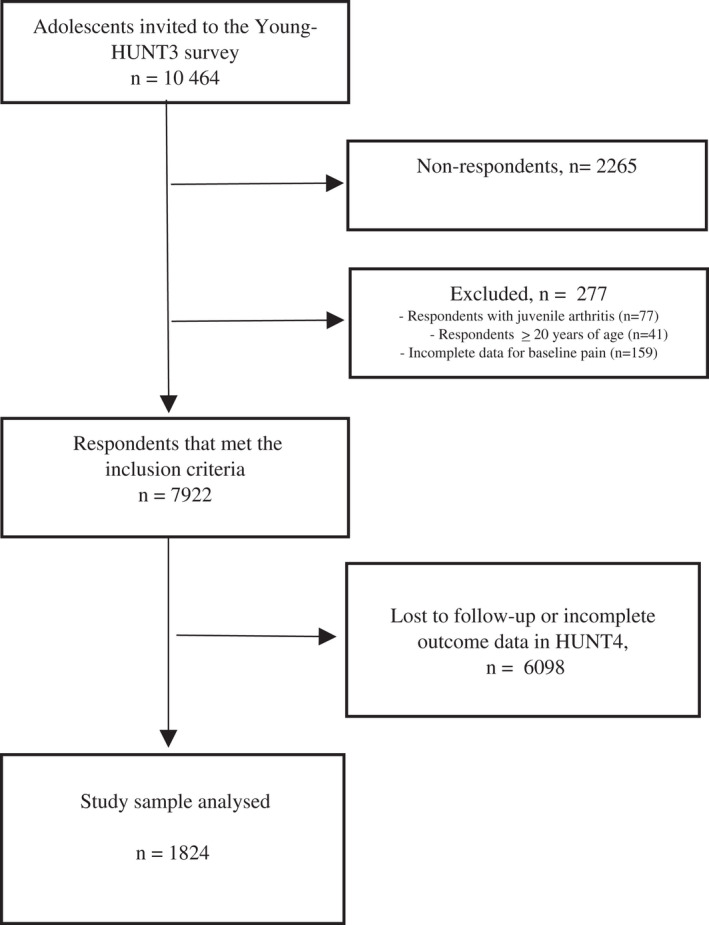
Flow chart of the study sample

Of 10,464 adolescents invited to participate in Young‐HUNT3, 8199 (78.4%) responded to the questionnaire. Of these, 277 participants were excluded in our study due to age ≥ 20 years, juvenile arthritis, or incomplete pain data at baseline, resulting in 7922 adolescents who met the inclusion criteria (Figure 1). All participants who were alive and still lived in Norway during the next follow‐up were invited to participate in HUNT4. A total of 1824 (23%) participants responded to the HUNT4 survey after 11 years and had complete outcome data for the present study. To investigate the exposure as both a potential “prognostic factor” and a potential “risk factor” in this study, the sample was divided in adolescents with musculoskeletal pain and adolescents without musculoskeletal pain at baseline, respectively.

The study protocol for this study was approved by the Regional Committee for Medical and Health Research Ethics (2019/517/REK Midt) in Norway, and the Norwegian Centre for Research Data (543422). All participants, and the parents or guardians of adolescents under the age of 16 years, signed a written informed consent form. The study protocol was published at ClinicalTrials.gov website (NCT04575974). The Strengthening the Reporting of Observational Studies in Epidemiology (STROBE) guidelines have been followed (von Elm et al., 2008) (Table S1).

### 2.2 Primary outcome

The primary outcome, *persistent musculoskeletal pain at follow‐up*, was defined as musculoskeletal pain lasting for at least three consecutive months (Treede et al., 2015), during the last year. It was measured by the question *“In the last year*, *have you had pain in muscles and joints that has lasted at least 3 consecutive months?”* presented together with a body chart. The participants who answered “*yes”* on this question were categorized as having persistent musculoskeletal pain at follow‐up.

### 2.3 Musculoskeletal pain assessment at baseline

*Musculoskeletal pain at baseline* was assessed by a questionnaire developed by Mikkelsson et al. (1996), which included a question of how often the participants had experienced pain unrelated to any known disease or acute injury during the past 3 months, accompanied by a body chart and a list of pain sites. The response alternatives were “seldom/never”, “about once a month”, “about once a week”, “several times a week” and “almost every day”. The participants who reported pain in the neck/shoulder, upper back, lower back, chest, left arm, right arm, left leg and/or right leg, once a month or more often during the last 3 months, were categorized as having musculoskeletal pain at baseline. The participants who reported musculoskeletal pain seldom/never the last 3 months were categorized as free of musculoskeletal pain at baseline.

### 2.4 Potential risk and prognostic factor

*The number of adverse lifestyle behaviours* was computed as a sum of six dichotomised individual behaviours, including low physical activity level, sleep problems, insufficient fruit and vegetables consumption, smoking, frequent alcohol intoxication (drunkenness) and illicit drug use, as described below. The variable was arranged as an ordinal variable and expressed as 0–1 (reference), 2, 3 and ≥4 adverse lifestyle behaviours in the model of individuals with musculoskeletal pain at baseline, and 0–1 (reference), 2 and ≥3 adverse lifestyle behaviours in the model of individuals without musculoskeletal pain at baseline, due to a low number of individuals with 4 or more adverse behaviours in the latter group.

*The physical activity level* was assessed with a question asking how many days a week the adolescents on average played sports or exercised outside school hours, to the point where they breathed heavily and/or got sweat. The question was adopted from the World Health Organization (WHO), Health Behaviour in School‐aged Children (HBSC) study (Currie et al., 2014). The questionnaire has been evaluated as reliable and correlates with cardiorespiratory fitness, especially for girls (Rangul et al., 2008). A cut‐off point at ≤ 1 day per week was used to classify low physical activity level, in accordance with previous research (Guddal et al., 2017; Rangul et al., 2008).

*Sleep problems* were assessed with the two questions “do you have trouble falling asleep at night?” and “do you wake up early and do not fall asleep again?” The response alternatives were “almost every night”, “often”, “sometimes” and “never”. Answering “often” or “almost every night” on one or both questions were classified as sleep problems. The questions were developed by the Norwegian Institute of Public Health for the Young‐HUNT study and have not been formally validated in adolescents.

*Fruit and vegetables consumption* was measured by questions of consumption frequency inspired by the Food Frequency Questionnaire (FFQ) used in the WHO HBSC study (Currie et al., 2014), which has been evaluated as reliable and has shown good agreement with a 24‐hour food behaviour checklist in adolescents (Vereecken & Maes, 2003). Answering once a day or less frequent on either the question of fruits consumption or the question of vegetables was classified as insufficient consumption, while reporting “several times a day” on both questions was considered a sufficient consumption, as this corresponds better to the recommendation of five servings of fruits and vegetables a day (World Health Organization, 2020).

*Smoking* was measured by the question “Do you smoke?” with the response alternatives “Yes, I smoke about … cigarettes daily”, “Yes, I smoke occasionally”, “No, I do not currently smoke, but earlier I smoked occasionally”, “No, I do not currently smoke, but earlier I smoked about … cigarettes daily” and “No, I do not smoke”. In the analyses, current daily and occasional smokers were defined as smokers.

*Frequent alcohol intoxication (drunkenness)* was measured by the questions “have you ever tried drinking alcohol?” and “have you ever been drinking so much alcohol that you felt intoxicated (drunk)?” The questions are adopted from the WHO HBSC study (Currie et al., 2014). Reporting to have been intoxicated >10 times were classified as frequent alcohol intoxication, in accordance with previous Young‐HUNT studies (Junker et al., 2017; Strandheim et al., 2009; Strandheim et al., 2010), while intoxicated ≤10 times or never been drinking alcohol were defined as infrequent episodes. As lifetime alcohol intoxication is higher in the older adolescent age groups, a lower threshold (2–3 episodes) of intoxications for the younger age group (13–15 years) was explored in sensitivity analyses.

*Illicit drug use* was measured by the question “have you ever tried hash, marijuana or other drugs?” Answering “yes” on this question was classified as illicit drug use. The question was developed by the Norwegian Institute of Public Health for the Young‐HUNT study and has not been formally validated in adolescents.

### 2.5 Background factors and potential confounders

Potential confounders were selected based on available literature and assumed theoretical associations. Age, sex, perceived family affluence (socioeconomic status) (Hanson & Chen, 2007; Huguet et al., 2016), psychological distress (Audrain‐McGovern et al., 2015; Crum et al., 2008; Huguet et al., 2016; Pourbordbari et al., 2019) and chronic diseases (Hestbaek et al., 2006b; Jones et al., 2006; Suris & Parera, 2005) were included as potential confounders in the analyses of both individuals with and without musculoskeletal pain at baseline. Additionally, number of pain sites (Pourbordbari et al., 2019) and pain impact on daily activities (Pate et al., 2020) were included in the analysis of individuals with musculoskeletal pain at baseline.

*Perceived family affluence* was used as a measure of socioeconomic status. It was measured by the question “how well off do you think your family is compared to most others?” with the response options “below others”, “average” and “above others”. The question is inspired by a measure in the WHO HBSC study (Currie et al., 2014). In the analyses, the response alternatives “average” and “above average” were merged.

*Psychological distress*, including symptoms of anxiety and depression experienced the last 2 weeks, was measured by a 5‐item version of the Hopkins Symptom Checklist (SCL‐5) (Derogatis et al., 1974; Strand et al., 2003). The five items were rated on a four‐point scale ranging from 1 (not at all bothered) to 4 (extremely bothered), and a mean score was calculated. This shortened version has been evaluated as reliable and is highly correlated with the longer versions, SCL‐25 and SCL‐10 (Strand et al., 2003), in which the latter one has been validated as a suitable instrument for detecting depressive symptoms in Norwegian adolescents (Haavet et al., 2011).

*Other chronic diseases* included reported asthma, diabetes, migraine and/or epilepsy. The diseases were measured with the question “has a doctor diagnosed you with:” followed by each of the diseases. The questions have not been formally validated in adolescents.

*Pain impact on daily activities at baseline* was measured by the question “Have the pain made it difficult for you to maintain daily activities?” with the subcategories “at school” and “in leisure time”. Adolescents with musculoskeletal pain who answered “yes” or “yes, sometimes” on either of the categories, combined with confirming pain in muscle or joints as the reason, were categorized as having musculoskeletal pain with impact on daily activities at baseline. The questions have not been formally validated in adolescents.

*Number of pain sites at baseline* included the number of musculoskeletal pain sites, headache, abdominal pain, and other pain, reported at least monthly the last 3 months at baseline. The variable was used as a dichotomised variable and categorised into ≤ two pain sites and ≥ three pain sites. A cut‐off of ≥ three pain sites was also included in the definitions of chronic multisite pain (Skrove et al., 2015) and diffuse idiopathic pain (Hoftun et al., 2011) in previous Young‐HUNT studies.

*Sex* (girl/boy) and *age* were measured. *BMI* (kg/m^2^) was calculated based on objective measurements of weight and height.

### 2.6 Statistical analyses

Variables were presented as counts and percentages if categorical, with mean and standard deviation (*SD*) if continuous and normally distributed, and with median and range if skewed. To assess possible selection bias, characteristics (e.g., sex, age, perceived family affluence) of respondents in the follow‐up survey were compared to characteristics of non‐respondents and respondents with incomplete outcome data, using independent T‐test for normally distributed continuous data, Mann–Whitney U test for skewed data and Chi‐square tests for categorical data.

Logistic regression analyses were used to estimate the association between the number of adverse lifestyle behaviours in adolescence and persistent musculoskeletal pain 11 years later. Individuals with musculoskeletal pain at baseline and individuals without musculoskeletal pain at baseline were analysed separately. In all models, persistent musculoskeletal pain was considered a binary outcome (presence of persistent musculoskeletal pain yes/no). The associations were investigated in crude and adjusted analyses and reported as odds ratios (ORs) with 95% confidence intervals (CIs). Adjustments were conducted in two blocks; firstly for sociodemographic factors only (age, sex, perceived family affluence), secondly for sociodemographic factors *and* health‐related factors (psychological distress, chronic diseases, pain impact on daily activities and number of pain sites [the latter two in the analyses of adolescents with musculoskeletal pain only]).

Sensitivity analyses were performed with lower threshold of alcohol intoxications (intoxicated 2–3 times) for the younger age group (13–15 years old), as lifetime alcohol intoxication is higher in the older adolescent age groups. To get an estimate of the individual contribution of each lifestyle behaviour, we conducted additional logistic regression analyses examining the association between individual lifestyle behaviours in adolescence and persistent musculoskeletal pain 11 years later, adjusted for sociodemographic and health factors.

For the regression analyses, missing data on exposure and confounders were replaced using a chained equation multiple imputation method, aiming to reduce potential selection bias due to missing data. All variables used in the regression analyses were used in the imputation model (White et al., 2011). To make the missing at random (MAR) assumption of the multiple imputation model more plausible, we also included some auxiliary variables (weekly musculoskeletal pain at baseline, BMI and current alcohol use [yes/no]) that were associated with variables to be imputed and/or with missingness in the variables (White et al., 2011). The outcome variable was included in the imputation model, but not imputed. Forty datasets were created. Complete case analyses were conducted for comparison.

*P*‐values <0.05 were considered statistically significant. All the analyses were conducted with STATA statistical software system, version 16 (StataCorp., 2019).

## 3 RESULTS

Characteristics of the study sample are presented in Table 1. At baseline, 65% of the adolescents reported musculoskeletal pain at least monthly the last 3 months. In the subsample of adolescents with musculoskeletal pain at baseline, there was a predominance of girls (69.7%), and the mean age at baseline was 16.1 (SD 1.8). In the subsample of adolescents without musculoskeletal pain at baseline there was 54.6% girls, and the mean age at baseline was 15.8 (SD 1.7) years.

**TABLE 1:** Baseline characteristics of the study sample

Characteristics	With musculoskeletal pain (*n* = 1186)	No musculoskeletal pain (*n* = 638)
Age, y, mean (*SD*)	16.1 (1.8)	15.8 (1.7)
Sex, girls, *n* (%)	827 (69.7)	348 (54.6)
Perceived family affluence, *n* (%)		
Average / above average	1016 (85.7)	567 (88.9)
Below average	104 (8.8)	39 (6.1)
Missing	66 (5.6)	32 (5.0)
Body mass index (BMI), median (min‐max)	21.6 (14.2–40.1)	21.0 (14.1–38.1)
Missing	63 (5.3)	41 (6.4)
Physical activity level, *n* (%)		
Moderate / high level	851 (71.8)	498 (78.1)
Low level	322 (27.2)	133 (20.8)
Missing	13 (1.1)	7 (1.1)
Servings of fruits and vegetables, *n* (%)		
Several times a day	121 (10.2)	86 (13.5)
Fruits and/or vegetables ≤ once a day	1047 (88.3)	538 (84.3)
Missing	18 (1.5)	14 (2.2)
Sleep problems, *n* (%)		
Sometimes /never	898 (75.7)	561 (87.9)
Often / almost every night	238 (20.1)	60 (9.4)
Missing	50 (4.2)	17 (2.7)
Smoking, yes, *n* (%)	182 (15.3)	37 (5.8)
Missing	28 (2.4)	5 (0.8)
Alcohol intoxication, *n* (%)		
0–10 times	689 (58.1)	390 (61.1)
> 10 times	305 (25.7)	117 (18.3)
Missing	192 (16.2)	131 (20.5)
Illicit drug use, yes, *n* (%)	46 (3.9)	7 (1.1)
Missing	9 (0.8)	3 (0.5)
Psychological distress^a^, median (min‐max)	1.6 (1–4)	1.2 (1–3)
Missing	26 (2.2)	15 (2.4)
Chronic diseases^b^, yes, *n* (%)	274 (23.1)	109 (17.1)
Missing	43 (3.6)	11 (1.7)
Number of adverse lifestyle behaviours^c^, *n* (%)		
0/1 adverse lifestyle behaviours	386 (32.5)	272 (42.6)
2 adverse lifestyle behaviours	297 (25.0)	146 (22.9)
3 adverse lifestyle behaviours	140 (11.8)	45 (7.1)
≥ 4 adverse lifestyle behaviours	95 (8.0)	16 (2.5)
Missing	268 (22.6)	159 (24.9)
Musculoskeletal pain at least weekly last 3 months, yes, *n* (%)	650 (54.8)	
Missing	24 (2.0)	
Musculoskeletal pain several times a week or daily last 3 months, yes, *n* (%)	371 (31.3)	
Missing	34 (2.9)	
Pain impact on daily activities, yes, *n* (%)	305 (25.7)	
Missing	41 (3.5)	
Number of pain sites, *n* (%)		
1–2 pain sites	278 (23.4)	
≥ 3 pain sites	872 (73.5)	
Missing	36 (3.0)	

^a^ Psychological distress, Hopkins Symptom Checklist – 5 (SCL‐5), 1–4.

^b^ Chronic diseases, include asthma, diabetes, migraine and epilepsy.

^c^ Number of adverse lifestyle behaviours, include low physical activity level, sleep problems, insufficient fruit/vegetables consumption, smoking, frequent alcohol intoxication and illicit drug use.

Those who were lost to follow‐up or with incomplete outcome data were more likely to be males, younger, to have an additional chronic disease, to have less psychological distress, to have a lower number of pain sites and to smoke compared to those with complete data at follow‐up (Table 2).

**TABLE 2:** Comparison of baseline characteristics of respondents and non‐respondents of the follow‐up study

Characteristics	Respondents (*n* = 1824)	Non‐respondents^d^ (*n* = 6098)	*P*‐value^e^
Age, y, mean (*SD*)	16.0 (1.8)	15.8 (1.7)	< 0.001
Sex, girls, *n* (%)	1175 (64.4)	2836 (46.5)	< 0.001
Perceived family affluence, *n* (%)			
Average / above average	1583 (91.7)	5153 (90.5)	0.13
Below average	143 (8.3)	541 (9.5)	
Missing, n	98	404	
Body mass index (BMI), median (min‐max)	21.4 (14.1–40.1)	21.5 (13.7–48.8)	0.38
Missing, n	104	468	
Physical activity level, *n* (%)			
Moderate / high level	1349 (74.8)	4589 (76.1)	0.26
Low level	455 (25.2)	1443 (23.9)	
Missing, n	20	66	
Servings of fruits and vegetables, *n* (%)			
Several times a day	207 (11.6)	730 (12.3)	0.39
Fruits and/or vegetables ≤ once a day	1585 (88.4)	5201 (87.7)	
Missing, n	32	167	
Sleep problems, *n* (%)			
Sometimes /never	1459 (83.0)	4746 (81.1)	0.06
Often / almost every night	298 (17.0)	1107 (18.9)	
Missing, n	67	245	
Smoking, yes, *n* (%)	219 (12.2)	924 (15.4)	0.001
Missing, n	33	96	
Alcohol intoxication, *n* (%)			
0–10 times	1079 (71.9)	3513 (72.1)	0.88
> 10 times	422 (28.1)	1360 (27.9)	
Missing, n	323	1225	
Illicit drug use, yes, *n* (%)	53 (2.9)	222 (3.7)	0.12
Missing, n	12	78	
Psychological distress^a^, median (min‐max)	1.4 (1–4)	1.4 (1–4)	0.01^f^
mean (SD)	1.52 (0.54)	1.49 (0.55)	
Missing, n	41	185	
Chronic diseases^b^, yes, *n* (%)	383 (21.6)	1407 (23.9)	0.047
Missing, n	54	215	
Number of adverse lifestyle behaviours^c^, *n* (%)			
0–1 adverse lifestyle behaviours	658 (47.1)	2085 (46.5)	0.052
2 adverse lifestyle behaviours	443 (31.7)	1310 (29.2)	
3 adverse lifestyle behaviours	185 (13.2)	643 (14.3)	
≥ 4 adverse lifestyle behaviours	111 (7.9)	446 (9.9)	
Missing, n	427	1614	
Musculoskeletal pain monthly last 3 months, yes, *n* (%)	1186 (65.0)	3827 (62.8)	0.08
Musculoskeletal pain weekly last 3 months, yes, *n* (%)	650 (36.1)	2079 (34.6)	0.24
Missing, n	24	91	
Musculoskeletal pain several times a week or daily last 3 months, yes, *n* (%)	371 (20.7)	1223 (20.6)	0.88
Missing, n	34	151	
Pain impact on daily activities, yes, *n* (%)	305 (17.1)	1069 (18.0)	0.36
Missing, n	41	175	
Number of pain sites, *n* (%)			
0–2	892 (50.1)	3162 (53.4)	0.02
≥ 3	888 (49.9)	2763 (46.6)	
Missing, n	44	173	

^a^ Psychological distress, Hopkins Symptom Checklist – 5 (SCL‐5), 1–4.

^b^ Chronic diseases, include asthma, diabetes, migraine and epilepsy.

^c^ Number of adverse lifestyle behaviours, include low physical activity level, sleep problems, insufficient fruit/vegetables consumption, smoking, frequent alcohol intoxication and illicit drug use.

^d^ Non‐respondents include both those who did not attend the follow‐up study at all, and those who did not answer the pain outcome of interest.

^e^ Categorical data analysed with Chi‐square test, normally distributed continuous data (age) analysed with T‐test, skewed data (BMI and psychological distress) analysed with Mann–Whitney U test.

^f^ The two distributions were both highly skewed and statistically significantly different despite the median being similar.

### 3.1 Lifestyle behaviour in adolescents *with musculoskeletal pain at baseline* and persistent musculoskeletal pain at follow‐up

In adolescents with musculoskeletal pain at baseline, a total of 36.6%, 39.7% and 45.3% of those reporting at least monthly, at least weekly and musculoskeletal pain several times a week or daily the last 3 months at baseline, respectively, reported persistent musculoskeletal pain at the 11‐year follow‐up. Adolescents with ≥4 adverse lifestyle behaviours at baseline had more than doubled odds of persistent musculoskeletal pain at the 11‐year follow‐up compared to those with 0 or 1 adverse lifestyle behaviour, adjusted for sociodemographic and health factors (OR 2.23, 95% CI 1.36, 3.66) (Table 3). Having 2 (OR 1.03, 95% CI 0.76, 1.38) and 3 adverse lifestyle behaviours (OR 1.06, 95% CI 0.71, 1.58) in adolescence were not statistically significantly associated with persistent musculoskeletal pain 11 years later, adjusted for sociodemographic and health factors (Table 3).

**TABLE 3:** Number of adverse lifestyle behaviours in adolescents *with musculoskeletal pain at baseline*, and persistent musculoskeletal pain at follow‐up (*N* = 1186)

Number of adverse lifestyle behaviours^a^	Exposed cases in the imputed data^b^	Crude	Adjusted 1^c^	Adjusted 2^d^
		OR (95% CI)	OR (95% CI)	OR (95% CI)
None or one	174–181	1 (Reference)	1 (Reference)	1 (Reference)
Two	133–140	1.07 (0.81, 1.41)	1.11 (0.83, 1.48)	1.03 (0.76, 1.38)
Three	62–68	1.20 (0.84, 1.73)	1.26 (0.86, 1.85)	1.06 (0.71, 1.58)
Four or more	53–57	**2.48 (1.60, 3.85)**	**2.70 (1.68, 4.35)**	**2.23 (1.36, 3.66)**

*Note*: Imputed data. Associations with a *p*‐value <0.05 in bold.

Abbreviations: CI, confidence interval; OR, odds ratio.

^a^ Number of adverse lifestyle behaviours include low physical activity level, sleep problems, insufficient fruit and vegetables consumption, smoking, frequent alcohol intoxication and illicit drug use. None or one adverse lifestyle behaviour, *n* = 520–534; two, *n* = 379–401; three, *n* = 166–175; four or more, *n* = 96–100 in the imputed datasets.

^b^ Number of participants with the specific number of lifestyle behaviours at baseline *and* persistent musculoskeletal pain at follow‐up, variation in numbers due to variation across imputed datasets.

^c^ Analyses adjusted for sociodemographic factors (age, sex and perceived family affluence).

^d^ Analyses adjusted for sociodemographic factors (age, sex and perceived family affluence) and health factors (psychological distress [SCL‐5], chronic diseases, number of pain sites and pain impact on daily activities).

Sensitivity analyses with lower thresholds of frequent alcohol intoxications of the younger age group revealed similar results as the main analyses (Table S2). Analyses of individual lifestyle behaviours, in the group of adolescents with baseline musculoskeletal pain, showed a statistically significant association between smoking in adolescence and persistent musculoskeletal pain at the 11‐year follow‐up, adjusted for sociodemographic and health factors (OR 1.74, 95% CI 1.24, 2.43). We found no statistically significant associations between low physical activity level (OR 1.20, 95% CI 0.91, 1.58), sleep problems (OR 1.10, 95% CI 0.80, 1.51), frequent alcohol intoxication (OR 1.0, 95% CI 0.72, 1.39), illicit drug use (OR 1.59, 95% CI 0.85, 2.95) or low intake of fruit/vegetables (OR 1.13, 95% CI 0.75, 1.70) in adolescence and persistent musculoskeletal pain after 11 years, adjusted for sociodemographic and health factors.

### 3.2 Lifestyle behaviour in adolescents *without musculoskeletal pain at baseline* and persistent musculoskeletal pain at follow‐up

In adolescents without musculoskeletal pain at baseline, 21.2% reported persistent musculoskeletal pain at the 11‐year follow‐up. In this group, having 2 (OR 1.02, 95% CI 0.64, 1.61) and ≥3 adverse lifestyle behaviours (OR 1.14, 95% CI 0.57, 2.30) at baseline, were not statistically significantly associated with persistent musculoskeletal pain at the 11‐year follow‐up, adjusted for sociodemographic and health factors (Table 4).

**TABLE 4:** Number of adverse lifestyle behaviours in adolescents *without musculoskeletal pain at baseline*, and persistent musculoskeletal pain at follow‐up (*N* = 638)

Number of adverse lifestyle behaviours^a^	Exposed cases in the imputed data^b^	Crude	Adjusted 1^c^	Adjusted 2^d^
		OR (95% CI)	OR (95% CI)	OR (95% CI)
None or one	80–83	1 (Reference)	1 (Reference)	1 (Reference)
Two	36–40	0.99 (0.63, 1.53)	1.04 (0.66, 1.65)	1.02 (0.64, 1.61)
Three or more	15–17	1.13 (0.61, 2.10)	1.32 (0.67, 2.61)	1.14 (0.57, 2.30)

*Note*: Imputed data.

Abbreviations: CI, confidence interval; OR, odds ratio.

^a^ Number of adverse lifestyle behaviours, include low physical activity level, sleep problems, insufficient fruit and vegetables consumption, smoking, frequent alcohol intoxication and illicit drug use. None or one adverse lifestyle behaviour, n = 385–390; two, n = 179–184; three or more, n = 67–70 in the imputed datasets.

^b^ Number of participants with the specific number of lifestyle behaviours at baseline *and* persistent musculoskeletal pain at follow‐up, variation in number due to variation across imputed datasets.

^c^ Analyses adjusted for sociodemographic factors (age, sex and perceived family affluence).

^d^ Analyses adjusted for sociodemographic factors (age, sex and perceived family affluence) and health factors (psychological distress [SCL‐5] and chronic diseases).

Sensitivity analyses with lower thresholds of frequent alcohol intoxications of the younger age group revealed similar results as the main analyses (Table S3). The analyses of individual lifestyle behaviours, in the group of adolescents without baseline musculoskeletal pain, showed no statistically significant associations between low physical activity level (OR 1.39, 95% CI 0.88, 2.20), sleep problems (OR 0.76, 95% CI 0.38, 1.51), frequent alcohol intoxication (OR 1.22, 95% CI 0.66, 2.28) or low intake of fruit/vegetables (OR 0.76, 95% CI 0.44, 1.32) in adolescence and persistent musculoskeletal pain at the 11‐ year follow‐up, adjusted for sociodemographic and health factors. The associations between smoking and illicit drug use in adolescence and persistent musculoskeletal pain at the 11‐year follow‐up were not estimated due to few exposed cases.

Contrasted with analyses of the imputed data, the results from the complete case analyses (Tables S4 and S5) indicated a slightly higher odds for persistent musculoskeletal pain at the 11‐year follow‐up for adolescents with baseline musculoskeletal pain and ≥4 adverse lifestyle behaviours compared to the reference group (0/1) (OR 2.77, 95% CI 1.60, 4.82 in complete cases [*N* = 811] vs. OR 2.23, 95% CI 1.36, 3.66 in imputed data [*N* = 1186]), and for adolescents without baseline musculoskeletal pain and ≥3 adverse lifestyle behaviours compared to the reference group (0/1) (OR 1.27, 95% CI 0.57, 2.82 in complete cases [*N* = 452] vs. OR 1.14, 95% CI 0.57, 2.30 in imputed data [*N* = 638]).

## 4 DISCUSSION

The aim of this study was to investigate whether an accumulation of adverse lifestyle behaviours in adolescence was associated with persistent musculoskeletal pain (pain duration ≥3 consecutive months the last year) 11 years later. The results showed, after adjustment for sociodemographic and health factors, that an accumulation of four or more adverse lifestyle behaviours in adolescents with musculoskeletal pain at baseline, including any combination of low physical activity level, sleep problems, insufficient fruit/vegetables consumption, smoking, frequent alcohol intoxication (drunkenness) and illicit drug use, was associated with persistent musculoskeletal pain 11 years later. An accumulation of adverse lifestyle behaviours in adolescents without musculoskeletal pain at baseline was in this study not associated with persistent musculoskeletal pain 11 years later.

To the best of our knowledge, this is the first study to investigate an accumulation of lifestyle behaviours in adolescence in relation to persistent musculoskeletal pain in adulthood, in a large population‐based study.

Our findings of an association between an accumulation of lifestyle behaviours and future persistent musculoskeletal pain in individuals with baseline pain, show similarities with findings from two previous studies on pain prognosis in adults. The studies reported that a combination of healthy lifestyle behaviours reduced the risk of long‐duration troublesome low back pain in women with occasional low back pain (Bohman et al., 2014), and long‐duration troublesome neck pain in men and women with occasional neck pain (Bohman et al., 2019). In our study, an accumulation of four or more adverse lifestyle behaviours in adolescence, irrespective of type, was associated with future persistent musculoskeletal pain, despite non‐significant associations for all individual lifestyle behaviours, except smoking. The results indicate the importance of examining a combination of lifestyle behaviours rather than only assessing individual behaviours in adolescents with musculoskeletal pain. Importantly, the mechanisms behind the association are not known. To increase this knowledge, we need further research on potential biological, psychological and social mechanisms.

The present study revealed no association between an accumulation of adverse lifestyle behaviours in adolescents without musculoskeletal pain at baseline and future development of persistent musculoskeletal pain. The highest exposure of accumulated behaviours investigated was three or more, due to few cases with more adverse behaviours in this group. Whether investigating a higher number of behaviours through a larger sample would affect the results is uncertain. Previous studies on adults have indicated that three or four healthy behaviours combined seems to be protective for future back pain (Pronk et al., 2010), long‐duration troublesome low back pain in men, and long‐duration troublesome neck pain in women (Skillgate et al., 2017). One potential reason for different results between these studies and our study might relate to the follow‐up periods, which in the previous studies were 2 and 4 years, respectively. Our 11 years follow‐up might involve changes in behaviours causing lack of association. Furthermore, the outcome in the previous studies captured spinal pain, while our study captured musculoskeletal pain in general. As the previous studies captured adults only, the difference in age groups might as well explain the different findings and indicates the importance of investigating risk factors in younger populations separately.

### 4.1 Strengths and limitations

The strengths of our study were the prospective design and the investigation of lifestyle behaviour and pain associations across important years of life for development of persistent musculoskeletal pain. A limitation of the study was the high proportion of participants lost to follow‐up or with missing outcome data (77%). The non‐respondents were more likely to be males, younger, to have an additional chronic disease, to have less psychological distress, to have a lower number of pain sites and to smoke compared to those with complete data at follow‐up. These differences are a potential source to selection bias. The non‐respondents also had slightly more adverse lifestyle behaviours compared to the respondents, but the difference was not statistically significant (P=0.052). It is unknown whether the non‐respondents had a different occurrence of the outcome at follow‐up. Another limitation was low statistical power in the analyses of adolescents without musculoskeletal pain due to few exposed cases. The time interval between baseline and follow‐up was 11 years. During this period lifestyle behaviour might have changed, which encourages us to be careful with the interpretation of the associations. More frequent follow‐ups could have given a better insight into trajectories of pain and lifestyle behaviours during the period.

All the lifestyle behaviours were self‐reported, which may have caused misclassifications. Misclassifications and loss of in‐depth information may also have occurred through dichotomisation of the variables. For example, both high and low physical activity levels have been associated with low back pain in previous research (Kędra et al., 2021). Through dichotomisation, a potential u‐shaped relationship was not taken into account (Heneweer et al., 2009). Furthermore, we classified ≥2–3 sessions of physical activities outside school (with moderate to vigorous intensity) as a “healthy” level, in accordance with the operationalisation of moderate to high level of physical activity used in previous research (Guddal et al., 2017; Rangul et al., 2008). However, as physical activity during school hours was not registered, it is uncertain whether all adolescents in this group reached the recommended level of 60 minutes daily (Bull et al., 2020). Sleep problems were included in the combined behaviour variable, although sleep problems might be a consequence rather than a choice of behaviour. We chose, however, to include it due to a potentially close relationship with the other lifestyle behaviours (Kwon et al., 2019; Lang et al., 2016), and its potential influence on health in youth (Shochat et al., 2014). To capturing an overall lifestyle behaviour, we included fruit/vegetables consumption, in accordance with previous research (Bohman et al., 2014; Bohman et al., 2019; Pronk et al., 2010; Skillgate et al., 2017). It should be noted that by including a solitary question covering fruit/vegetables consumption, only a limited part of diet behaviour was explored.

Pain intensity, which has been reported as a potential prognostic factor in clinical settings (Pate et al., 2020) was not adjusted for as we were limited to variables available in the HUNT study. Our results are based on a sample derived from community and may not be applicable to adolescents with musculoskeletal pain who are seeking health care.

### 4.2 Implications

Our findings of an association between an accumulation of adverse lifestyle behaviours and future persistent musculoskeletal pain, in individuals with baseline pain, might have implications both on public health strategies and clinical approach. We suggest that lifestyle behaviours should be assessed in adolescents with musculoskeletal pain, and that accumulation of multiple adverse lifestyle behaviours should be considered in future health care and secondary prevention interventions. This is of particular interest given the additional influence of lifestyle behaviour on several other health conditions (Loewen et al., 2019; Tsai et al., 2020; Zhang, Pan, Chen, Cao, et al., 2020; Zhang, Pan, Chen, Xia, et al., 2020). Exploration of underlying and mediating mechanisms of the association, as well as development of interventions targeting multiple lifestyle behaviours in adolescents with musculoskeletal pain is needed. Our analyses of a possible association between an accumulation of adverse lifestyle behaviours and later persistent musculoskeletal pain in individuals without musculoskeletal pain at baseline, should be repeated in a large sample with more frequent follow‐ups.

## 5 CONCLUSION

In this study, an accumulation of adverse lifestyle behaviours in adolescents with musculoskeletal pain at baseline was associated with persistent musculoskeletal pain 11 years later, but not in adolescents without musculoskeletal pain at baseline. Accumulation of multiple adverse lifestyle behaviours should be considered in health care and future research of adolescents and young adults with musculoskeletal pain.

## AUTHOR CONTRIBUTION

KS designed the study, conducted the analyses, interpreted the results, and drafted and critically revised the manuscript for intellectual content. MG, HJ, KRR, MCS, ES and BEØ designed the study, provided substantially contribution to the interpretation of results, and critically revised the manuscript for important intellectual content. All authors approved the submitted version of the manuscript.

## FUNDING INFORMATION

This study was supported by the Norwegian Fund for Post‐Graduate Training in Physiotherapy (grant number 105597). The funder organization played no role in designing the study, in the collection, analysing or interpretation of the data, or in writing the manuscript.

## CONFLICT OF INTEREST

We declare no conflicts of interest.

## Supporting information


Tables S1‐S5
